# Partitioning of Antioxidants in Edible Oil–Water Binary Systems and in Oil-in-Water Emulsions

**DOI:** 10.3390/antiox12040828

**Published:** 2023-03-28

**Authors:** Sonia Losada-Barreiro, Fátima Paiva-Martins, Carlos Bravo-Díaz

**Affiliations:** 1 Departamento Química-Física, Facultad de Química, Universidade de Vigo, 36310 Vigo, Spain; 2 REQUIMTE-LAQV, Departamento de Química e Bioquímica, Faculdade de Ciências, Universidade do Porto, 4169-007 Porto, Portugal

**Keywords:** partition constant, antioxidant, edible oils, emulsion, intermolecular interactions

## Abstract

In recent years, partitioning of antioxidants in oil–water two-phase systems has received great interest because of their potential in the downstream processing of biomolecules, their benefits in health, and because partition constant values between water and model organic solvents are closely related to important biological and pharmaceutical properties such as bioavailability, passive transport, membrane permeability, and metabolism. Partitioning is also of general interest in the oil industry. Edible oils such as olive oil contain a variety of bioactive components that, depending on their partition constants, end up in an aqueous phase when extracted from olive fruits. Frequently, waste waters are subsequently discarded, but their recovery would allow for obtaining extracts with antioxidant and/or biological activities, adding commercial value to the wastes and, at the same time, would allow for minimizing environmental risks. Thus, given the importance of partitioning antioxidants, in this manuscript, we review the background theory necessary to derive the relevant equations necessary to describe, quantitatively, the partitioning of antioxidants (and, in general, other drugs) and the common methods for determining their partition constants in both binary (*P*_W_^OIL^) and multiphasic systems composed with edible oils. We also include some discussion on the usefulness (or not) of extrapolating the widely employed octanol–water partition constant (*P*_W_^OCT^) values to predict *P*_W_^OIL^ values as well as on the effects of acidity and temperature on their distributions. Finally, there is a brief section discussing the importance of partitioning in lipidic oil-in-water emulsions, where two partition constants, that between the oil-interfacial, *P*_O_^I^, and that between aqueous-interfacial, *P*_w_^I^, regions, which are needed to describe the partitioning of antioxidants, and whose values cannot be predicted from the *P*_W_^OIL^ or the *P*_W_^OCT^ ones.

## 1. Introduction

The increasing consumer demand for natural food additives and ingredients has stimulated the search for different sources of antioxidants (AOs) [1,2,3,4,5]. An enormous number of plants and biomass wastes, including those from natural food sources, have been screened as potential antioxidants [6]. Most of these studies have focused on the identification of bioactive components in order to reach a (more) sustainable world by searching for new bio-renewable sources for valuable components [7] and adding value to waste [8,9]. These bio-renewable sources are of interest for food, cosmetic, pharmaceutical, and medicinal industries, and have the additional advantage of being largely available, widely distributed, and inexpensive, particularly when their uncontrolled disposal would create serious environmental problems, especially when they are geographically concentrated [1,10,11,12].

In recent years, there has been increased concern regarding the reduction of food loss and waste, and consumers have been demanding more sustainable food systems. Alternative pathways for food waste management include the valorization of by-products as a source of phenolic compounds for functional food formulations [13]. This is the case for phenolic antioxidants, an important group of compounds with enormous potential as a result of their beneficial properties in health and in the prevention of the oxidative deterioration of edible oils [14]. Phenolic extracts from natural sources also exhibit a wide range of physiological properties, including anti-thrombotic, anti-atherogenic, anti-allergenic, anti-inflammatory, anti-microbial, vasodilatory, and cardioprotective effects [4,15]. They are present in a number of agro-industrial by-products (husks, peels, seeds, etc.), fruit peels, olive mill waste, olive leaves, and residue from grapes and winemaking are all considered important natural sources of antioxidants [4]. In particular, olive mill wastewaters have been identified as a promising source of antioxidants to retard lipid oxidation in fish oil-enriched food products, and the use of active packaging films containing antioxidants is being explored [16,17,18,19].

**Figure 1:**
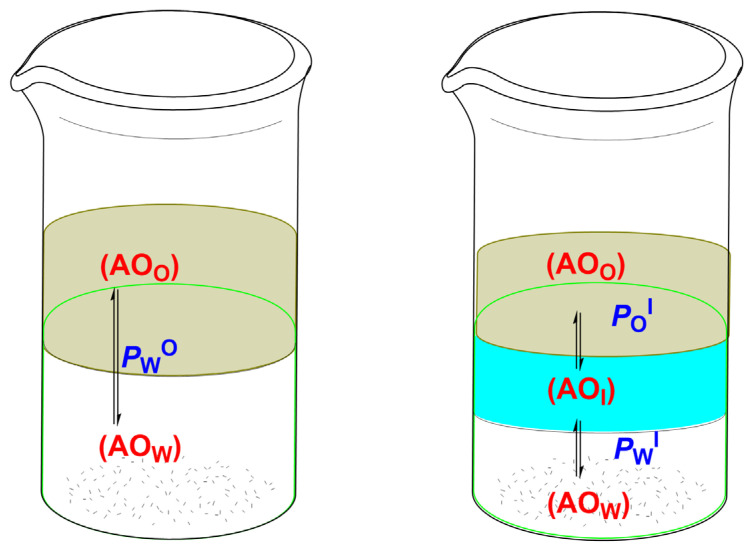
Basic representation of the distribution of an antioxidant (AO) in a binary oil–water system (**left**) and in an emulsified system (**right**) between the oil (O), interfacial (I), and aqueous (W) regions. The solvation spheres of the antioxidant in the different regions are not displayed for the sake of clarity.

Partitioning is, thus, an important phenomenon not only in medicinal chemistry and drug design, but also in the recovery of antioxidants from wastewaters, biomass, etc. Common procedures for the extraction of antioxidants from wastewaters require, among others, the use of aqueous and organic solvents [20], where antioxidants are partitioned according to their hydrophobicity [21,22]. Determining the partition constant values of antioxidants (and, in general, of other compounds) in binary oil–water mixtures and in multiphasic systems such as emulsions is, thus, of great interest to the industry for, among others, the following reasons:

(1) The partition constant value of a molecule (e.g., antioxidant (AO)) is frequently employed as a “measure” of its relative affinity to be incorporated in a given region (Figure 1), and its “tendency” to be dissolved in such a region depends on the value and sign of the Gibbs free energy of transfer from one region to another one (Δ*G*_transfer_ < 0) [23,24,25,26,27]. In binary oil–water mixtures, the distribution of an antioxidant can be described by one partition constant *P*_W_^O^, while in emulsified systems, two partition constants are needed, *P*_W_^I^ (that between the aqueous and interfacial regions) and *P*_O_^I^ (that between the oil and interfacial regions), Figure 1.

Once extracted from the raw material, antioxidants (or their extracts) are added to different delivery systems that range from simple aqueous solutions to complex emulsified systems, and where they partition between the different phases/regions, thus affecting their biological or chemical activity [5,28,29,30,31,32].

(2) Research work carried out during recent years has shown that olive oil–water partition constants are better correlated with drug efficiency and their partitioning into human tissues [33,34,35,36]. For instance, Poulin et al. [37] showed that the partition constants determined in olive oil–water systems (*P*_W_^OIL^) are clearly superior to those determined in octanol/water systems (*P*_W_^OCT^) for predicting adipose tissue plasma partition coefficients, because olive oil better represents the lipid composition of the adipose tissue content, which is primarily composed of triglycerides. In addition, antioxidants need to pass through lipid membranes to reach their targets, and these lipid membranes can also cause them to partition [38].

(3) Partition constant values depend on the solvation properties of a particular region, usually computed in terms of hydration/solvation free energies [39,40]. Thus, one can obtain insights on the molecular environments of antioxidants by determining their partition constants in edible oil–water systems and, by extrapolation, to determine their bioconcentrations in living systems such as fish [34,35].

(4) In multiphasic systems such as emulsions, antioxidants are added to minimize the oxidation of lipids. The partition constant values between the various regions define their distribution within the system, and such distribution is crucial for assessing their efficiency in inhibiting lipid peroxidation as mass transfers affect chemical reactivity because the “local” or “effective” concentrations of antioxidants, which depend on the partitioning of the reactant, may be orders of magnitude different from one region to the other and much higher or lower than the stoichiometric concentrations [41,42,43,44,45].

(5) Partitioning of antioxidants is also crucial for developing new encapsulation systems employed to protect them and to deliver them in a controlled manner to target environments. These technologies have attracted great interest in preparing functional foods, nutraceuticals, and pharmaceutical formulations because of the important health benefits that antioxidants have in the prevention of human diseases [4,30,46,47].

(6) A literature search carried out by employing Scifinder (CAS, accessed on 3 March 2023) revealed that in the 2012–2023 period, the number of published papers containing the searching criteria “partitioning ” were higher than 5000; however, it decreased significantly to only 236 when the criteria “partitioning + antioxidants” were used. The number of published papers in the period was even lower (98) when searching for publications containing emulsion (partitioning + antioxidant + emulsion) or the keywords “partitioning + antioxidant + oil-in-water” (60 papers). These numbers reveal that much of the published works on partitioning employ molecules other than antioxidants and thus reinforce the need for a review paper updating and enhancing the published information on antioxidant partitioning.

We noticed, when searching in the literature, that the terms “partition constant” and “partition coefficient” are often used, sometimes leading to confusion regarding the proper use of these terms. IUPAC recommendations [48] suggest that *P*_W_^O^ should be better defined as “partition ratio”; however, in many scientific discussions and in specialized literature, the term partition constant or partition coefficient is commonly employed [21,23,49]. Thus, for convenience and for the sake of clarity, hereafter we will employ the term “partition constant” to describe the equilibrium partitioning of neutral antioxidants between two (essentially) immiscible phases, namely oil (O) and water (W), so that the ratio between the effective concentrations in each of the two phases, i.e., the *P*_W_^O^ value, is a concentration-independent constant at given experimental conditions. Examples of these systems include edible oil–water and the widely employed *n*-octanol–water binary systems, where partitioning molecules may be present both as neutral and charged species. Some authors employ the term *D*_W_^O^ instead of *P*_W_^O^ to denote the apparent partition constants in the pH range where ionic species coming from ionizable drugs are present [25,50]. For the sake of clarity, consistency, and simplicity, in these cases, we will use the term *P*_W_^O^ (app). Other terms that can also be found in the specialized literature such as “distribution constants”, “distribution coefficients”, or “distribution ratio” are less common and will not be employed here.

## 2. Physicochemical Basis of Partitioning: Balance of Intermolecular Interactions

Partitioning is intimately related to the concept of hydrophobicity, which is commonly understood to be the tendency of non-polar molecules to reduce its contact with polar molecules, such as water, but to increase that with other non-polar molecules [51]. Daily manifestations include the simple observable macroscopic phenomena of immiscibility between oil and water and constitute the foundations of some modern analytical techniques such as chromatographic separation. A central point in the study of partitioning phenomena are the intermolecular forces: the same set of forces and interactions that partition a molecule between polar and hydrophobic solvent phases, i.e., determines its hydrophobicity, can also be employed to explain the physical states of matter and even biological interactions, including small molecule binding and protein folding.

To fully understand the partitioning phenomena, its significance, and, most importantly, to be able to predict partition constant values in different binary oil–water systems, we first must be aware of the different kinds of interactions that may exist between solutes, solute–solvents, and their relative strengths in both phases. It will be the balance of all these attractive and repulsive forces that, in the end, will dictate the relative affinity of the antioxidant (or other molecules of interest) for the two competing phases. We therefore need to identify the nature, number, and position of functional groups (e.g., -OH, -CH_2_-, and -COOH) present in the antioxidants that partition and in the solvents to identify the kinds of intermolecular forces (or interactions) that are “formed” or “broken” during the partitioning process. Attractive forces between molecules stem from uneven electron distributions, deserving some discussion considering the regions of the solute and solvent molecules where electron-rich or electron-deficient molecules may be located [52,53].

The process of transfer of an antioxidant (AO; or, in general, any other molecule) from within the oil phase to within the aqueous phase (or vice versa) implies that intermolecular attractions are “formed” and “broken” during the exchange. These interactions are much weaker (1–12 kJ/mol) than covalent bond strengths (50–300 kJ/mol), but they are able to control critical characteristics of matter such as the boiling and melting points, vapor pressure, and viscosity. For the sake of simplicity, hereafter, we will consider that there is not physical restriction to the transfer of antioxidants between phases, and that this partitioning is governed exclusively by the thermal motion of molecules so that partitioning takes place essentially at the time of bulk mixing solutes and solvents.

The resulting interactions between uncharged solutes or solvent molecules arise from the attractive or repulsive electrostatic forces between electron deficient regions in a molecule that are attracted by electron-rich or repelled by electron-deficient counterparts in neighboring molecules. The overall affinity of molecules for each other results from the balance of all these attractions/repulsions. A summary of the main intermolecular forces is given in Table 1 and further details on their origin, nature, and energetics can be found elsewhere [54,55].

For descriptive purposes, the intermolecular forces can be divided in two main groups:

(1) Nonspecific interactions, which are those that exist between any kind of molecules, no matter their chemical structures. They are globally known as the van der Walls forces (vdW) and include dipole–dipole interactions (Keeson), dipole–induced dipole interactions (Debye), and induced dipole–induced dipole interactions resulting from uneven electron distributions over time (London dispersive forces) [55].

The dipole–dipole (Keeson) interactions arise from the electrostatic interaction between molecules with permanent dipoles that makes them orient so that the dipoles face each other so as to minimize repulsive forces, that is, electrostatic attractive forces between the positive end of one polar molecule and the negative end of another polar molecule. The strengths of these attractions are proportional to the product of the permanent dipole moments of the two interacting molecules and depend on the orientation of the interacting partners, ranging from 1 to 5 kJ per mole. They are, therefore, much weaker than ionic or covalent bonds and have a significant effect only when the molecules involved are close to each other.

Hydrogen bonding is a special case of dipole–dipole interaction that takes place between the hydrogen atom in polar N-H, O-H, or F-H bonds and electronegative atoms such as O, N, or F. Hydrogen bonding (H-bonding) is a highly directional, noncovalent interaction, present in many organic molecules that is, notably, responsible for supramolecular ordering in biological systems, because proteins and nucleic acids are composed of numerous -NH and -OH groups that can donate hydrogen bonds, and C=O and other groups that can accept them. These forces are responsible, for example, for the microscopic structure of water [55] or for the strength of cellulose fibers.

Dipole–induced dipole interactions (Debye) occur when a polar molecule with a permanent dipole (e.g., molecules containing bonded atoms with different electronegativities (e.g., O-H)) disturbs the arrangement of electrons in nearby non-polar molecules, inducing a dipole.

The London dispersion forces (induced dipole–induced dipole attractive forces) are related to the repellency of electrons of non-polar molecules when they approach each other, resulting in the formation of two induced dipoles leading to intermolecular attractions. At any instant, the electron distribution may be unsymmetrical and hence produce an instantaneous dipole. This can cause an induced transient dipole in the neighboring molecule and can cause the molecules to be attracted. The London dispersion force is, by far, the weakest intermolecular force. In spite of this, it is very important because it is universal and causes, for instance, non-polar substances to be liquids or solids if the temperature is lowered sufficiently [55].

When electron donor–acceptor interactions are negligible, London dispersive forces are the main contributors to the overall attraction of many molecules to their neighbors. If molecules show stronger interactions than those expected from these universal interactions, then it means that other intermolecular forces make an important contribution to the overall balance. It is worth noting that the London interactions are universal because their origin arises from momentary displacements of electrons within the structure of the molecule (in the order of the femtosecond time scale), so that there is a continuous presence of short-lived dipoles in the structure, and these fleeting dipoles are felt by neighboring molecules whose electrons react in a complementary fashion, thus inducing instant dipoles [55].

A particular and important type of dispersion force from van der Waals forces is π–π stacking interactions. As the electron distributions in aromatic systems are relatively easily distorted, they can engage in atypically strong induced dipole–induced dipole interactions called π–π stacking interactions. These interactions are named this because they occur when the planes of aromatic rings are stacked parallel to one another. This parallel stacking can occur in either a sandwich or a displaced stacking arrangement. Noncovalent interactions involving aromatic rings are pivotal to protein–ligand recognition and concomitantly to drug design. Indeed, the vast majority of X-ray crystal structures of protein complexes with small molecules reveal bonding interactions involving aromatic amino acid side chains of the receptor and/or aromatic and heteroaromatic rings of the ligand.

(2) Specific (or polar) interactions that result from molecular structures that enable attractions between permanent electron-poor parts of a molecule (e.g., H—attached to oxygen) and electron-rich sites in another molecule (e.g., non-bonded electrons of atoms such as O and N). These polar interactions are, thus, only possible when molecules bear complementary structural moieties: one moiety acts as an electron donor (H-acceptor) and the other one as an electron acceptor (H-donor). Both electron donor–acceptor (EDA) and hydrogen donor–acceptor (HAD) are terms that are widely used in the literature. Hydrogen acceptors are electronegative atoms (N, O, or F) of a neighboring molecule or ion that contain lone electron pairs. Typical H-donor groups are molecular moieties containing electronegative atoms such as N, O, and F that are covalently bonded to a hydrogen atom because they pull the covalently bonded electron pair closer to its nucleus, but away from the H atom. The H atom, consequently, has a partial positive charge, creating a dipole–dipole attraction between the hydrogen atom bonded to the donor and the lone electron pair of the acceptor [55].

## 3. Energetics of Partitioning: Thermodynamic Equations

The distribution of a solute between two phases is an equilibrium condition that can be described in terms of the energetics (thermodynamics) of the process [49,56]. Several situations can be found depending on the nature of the two phases involved: liquid–liquid, liquid–solid, gas–liquid, and gas–solid. Here, we focus on liquid–liquid biphasic systems because they are the basis for solvent extraction, constituting one of the most important extraction methods commonly employed to separate compounds based on their relative solubilities in the immiscible liquids [22].

To simplify the mathematics as much as possible, several reasonable assumptions were made. We assumed that (i) the antioxidant of interest is chemically stable, partitions between two immiscible bulk phases that are in contact with each other at a given temperature and pressure, and does not react with any component of the solvent mixture. (ii) The oil and water phases are at equilibrium with each other with respect to the amounts of all chemical species present in each phase and that there is no physical barrier that prevents the free movement of molecules between them, as illustrated in Figure 1. If the phases (e.g., oil and water) are not strictly immiscible, it is assumed that each phase is saturated with the molecules of the other one after bulk mixing the phases. (iii) We presumed, as a first approach, that when a small amount of an antioxidant C is added to the binary mixture, (a) the bulk properties of both phases are not significantly disturbed by the introduction of the antioxidant molecules and (b) the solubility limit of the antioxidant in any of the phases is not reached.

Under these conditions, the chemical potentials of the antioxidant in water (*µ*_C_^W^) and oil (*µ*_C_^O^) are given by Equations (1) and (2), respectively, where R is the universal gas constant [21,23,49,57]. Equations (1) and (2) show that the chemical potentials depend on the activity of the solute (*a*_C_ = **γ**_C_C_C_, where γ_C_ and C_c_ stand for the activity coefficient and the concentration of the antioxidant in units mol/L, respectively), and when the concentrations of the solute are low enough to make solute intermolecular interactions negligible (i.e., dilute solutions), it is safe to use the concentrations instead of activities (i.e., γ_I_ ≈ 1) [21,49].

(1) $$\mu_{C}^{W}=\mu_{C}^{0,W}+RT\ln\;a_{C}^{W}$$

(2) $$\mu_{C}^{O}=\mu_{C}^{0,O}+RT\ln\;a_{C}^{O}$$

The Gibbs free energy change accompanying the transfer of the antioxidant from one phase (e.g., water) to the other phase (e.g., oil) in equilibrium with each other, at a constant temperature *T* and pressure, is given by Equation (3), where Δ*G*, Δ*H*, and Δ*S* are the Gibbs free energy, enthalpy, and entropy changes, respectively, and *T* is the absolute temperature.

(3) $$\Delta G^{0,W\to O}{=\Delta H}^{0,W\to O}-{\;T\Delta S}^{0,W\to O}=\mu_{C}^{0,O}-\mu_{C}^{0,W}$$

Thus, when the change in Gibbs free energy is negative, the antioxidant is transferred spontaneously from the water to the oil, otherwise it is not, and needs some energy input to be transferred after some time. Assuming that the transfer process is spontaneous, after a short time, some antioxidant molecules are transferred from one phase to the other until the equilibrium is attained, so that the chemical potentials of the antioxidant in the oil and water phases are equal each other. At equilibrium, Δ*G* = 0 and Equation (4) can be derived, where (C_o_) and (C_W_) are the effective concentrations of the antioxidant C in the oil and water phases (moles of the antioxidant *C* per liter of phase volume), respectively. Further details on the assumptions and equations involved can be found elsewhere [21,27,58].

The partition coefficient *P*_W_^O^ is, thus, defined as the ratio of the effective concentrations of the solute in the oil and water phases (moles of antioxidant per unit volume of each phase). In some textbooks, the partition constants are expressed as mole fractions, and one can easily convert mole fractions to molar concentrations, bearing in mind the molar volume of the mixture or solution, C = x_i_ (mol i/total mol)/V_M_ (L (total mol)^−1^) assuming that the volumes are additive to a first approximation [27,49].

(4) $$\Delta G^{0,W\to O}=-\;RT\ln\frac{{(C}_{O})}{{(C}_{W})}=-\;RT\ln P_{w}^{O}$$

To avoid future (potential) misunderstandings, we would like to recall here that the effective concentrations of the antioxidant in each of the phases, e.g., (C_O_) and (C_W_), are, in general, different from the stoichiometric concentration, [C_T_], which is expressed in moles per liter of the total volume solution (oil + water). To avoid misconceptions, and for the sake of clarity, hereafter, stoichiometric concentrations (in moles per liter of total volume of the oil–water mixture) will be indicated by the commonly employed brackets [ ] meanwhile parentheses ( ) will indicate molar concentrations in moles per liter of a particular phase.

Several factors contribute to *P*_W_^O^ values, including the molar volume of the solute, solvation parameters (H-bond donor acidity/acceptor basicity), and polarizability (orientation and induction forces). All of these contributions affect the energy costs required for exposing non polar solutes to water molecules and create hydrophobic–water contacts. It has also been argued that the formation of ion pairs between the ionized antioxidant and the strong electrolytes present in the aqueous phase also need to be considered. Equations for limiting cases, where the formation of ion pairs is negligible, where there is a high tendency to form ion pairs and where no ion pair and no dissociation takes place in the oily region have been derived. Certainly, comparisons of the three models may help to better understand the fate of ionizable antioxidants and the interested reader can obtain further information on the various possibilities elsewhere [25,50].

## 4. Methods to Measure Partition Constants

**Figure 2:**
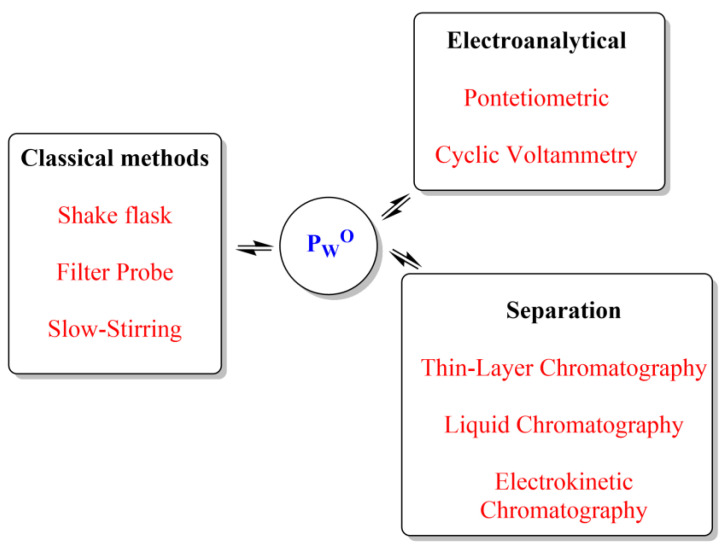
Common experimental methods employed to determine the partition constant values.

Numerous methods are available to estimate the partition constants of molecules of interest in binary oil–water systems, all of them having strengths and weakness, and limitations regarding the nature of the compounds that can be used. Here, we describe some of the most common methods, and for convenience, we have divided them into those requiring laboratory set-ups (experimental methods, Figure 2), and those that employ in silico calculations (computer software). It is necessary to recall that computer methods are always estimative and that the estimated values need to always be corroborated with experimental, accurately determined, *P*_W_^O^ values.

Table 2 summarizes some of the most common experimental and computer packages employed. We will only describe some of them briefly and the interested reader is referred to specialized reports [23,27,59,60,61].

### 4.1. Experimental Methods

Most likely, the simplest (and traditional) method to determine *P*_W_^O^ is the shake-flask method [22,23], where an oil–water mixture is spiked with the antioxidant and shaken at constant temperature for some time until equilibrium is achieved [49]. Aliquots of the organic and aqueous phases are then extracted or, otherwise, phases are separated (by centrifugation, for example) and the concentration of the chemical in each phase is determined by employing any suitable analytical technique from previously prepared calibration plots. The method presents some drawbacks, mainly concerned with the necessary time to carry out the experiment and the lack of reliability when employing very hydrophobic or hydrophilic chemicals. Deviations may also occur when the tested chemical dissociates and the partition constant becomes pH and/or concentration dependent. The main advantage is its experimental simplicity and reliability [22,27].

Other (indirect) methods are based on the partition of antioxidants between a non-polar liquid organic phase and a polar aqueous phase in an HPLC column [22,62]. This is probably the most rapid and accurate method, but requires that the laboratory possesses a reverse-phase HPLC, which is a relatively expensive instrument—indeed much more than the simple separator funnels employed in the shake-flask method. In addition, solvents for the mobile phase are required and the results may be affected by the operating retention mechanisms. Frequently, multivariate techniques and molecular descriptors might need to be used for antioxidants with quite different chemical structures from that of the reference substances employed. Variations of the RP-HPLC method, including micellar electrokinetic chromatography and counter-current chromatography, have also been employed to determine partition constants [63,64,65].

### 4.2. Computational Methods: Extrathermodynamic Approaches Based on Linear Free Energy Relationships (LFERs) to Predict and/or to Evaluate Partition Coefficients

The importance of predicting log *P*_W_^O^ values arises because they are usually taken as a measure of the lipophilicity of the antioxidants, and food, pharmaceutical, and biological events depend on the lipophilic characteristics of the chemical species involved. Despite its importance, it is rather common that researchers find situations in which the partitioning behavior of a compound between organic matter (in its broad sense) and water is needed, but either cannot be easily determined experimentally or, for any other reason, some of the data required are not available. In these cases it is thus important to develop predictive methods to allow researchers to obtain estimates of the partition coefficients [23,24,58,66].

The basic idea behind the most common approaches used for predicting partition constants is to express the (unknown) free energy of transfer of the molecule of interest Δ*G*_transfer_ in the two-phase system by one or several other known free energy terms, chosen in a way that can be linearly related to Δ*G*_transfer_. The relationships between the unknown Δ*G*_transfer_ and the known free energy terms are usually called linear free energy relationships (LFERs; Table 3) [23,24,57,66]. Such relationships need to take into consideration the molecular interactions that exit between solute molecules and solute–solvent molecules.

Pretty good correlations can be obtained when considering similar systems, particularly when choosing groups of compounds that undergo the same type of interactions in a given phase. However, as we will see later (Section 5), it should not be surprising that poor relationships are found when trying to relate partition constants of a series of compounds of different polarities between two systems that contain phases with different solvent properties, for example, when attempting to predict partition constant values in edible oil–water binary mixtures from those obtained in octanol–water systems [67,68].

A second, conceptually different approach, assumes that Δ*G* of transfer for the whole molecule can be expressed by the sum (linear combination) of the terms describing the free energy of transfer of the various parts of the molecules [69]. To describe intramolecular interactions between different parts of the molecule that cannot be accounted for when considering the transfer of the isolated parts, it is necessary to include special interaction terms. The major advantage of this approach is that it allows one to estimate a partition constant based solely on the structure of the considered compound, and good results can be anticipated particularly in those cases where predictions of the partition constant of a structurally similar compound is known, so that only the contributions of the parts that are different between the two compounds have to be added and/or subtracted.

The most advanced and most widely used method that is based on this concept is the structural group contribution method for estimating partition constants. The method was first proposed by Hansch and Leo in a seminal paper published in 1971 [56], where they explain the fundamentals of the partitioning phenomena, providing detailed descriptions of the theory, the various uses of the partition constants, and a very comprehensive tabulation of a large number of *P*_W_^O^ values for a variety of substances. However, most interestingly, they also provided a discussion on additive–constitutive properties describing the use of various known linear-free relationships (e.g., Hammet equation) to calculate partitioning free energy and the various stereoelectronic effects on the partition constant values. The importance of the quantification of hydrophobic interactions energies has been, and still is, the key to drug design projects, as well as the routines included in many software currently employed in predictive studies and in computational chemistry [49,56,58].

#### Fragment-Based, Atom-Based, and Molecular Methods for Estimating Partition Constants

Theoretical prediction of the *P*_W_^O^ values is a convenient procedure because it is time-saving with respect to experimental, time-consuming methods and because in many instances, the number of existing experimental data are negligible compared with the number of chemicals for which it is necessary. Most of the models that employ current software packages are based on the fragmentation of the compound of interest in substructures and/or in the calculation properties of each fragment. Several statistical parameters are considered to assess the predictive values of *P*_W_^O^, and most of them are based on the use of quantitative structure–reactivity relationship (QSARs) models, but some structural or physicochemical properties such as surface-activity, ionization, and poor solubility make it difficult to determine *P*_W_^O^ values accurately, or perhaps the compound analyzed is chemically unstable and undergoes rapid degradation [70,71]. We will describe some of them briefly.

Fragment-based computational methods to estimate *P*_W_^O^ values were first proposed by Mannhold [72]. According to this approach, the molecule is essentially broken down into fragments so that the actual *P*_W_^O^ value can be calculated as the sum of the contributions of each fragment. However, correction factors are usually needed to compensate for intramolecular interactions because the various molecular environments may affect the effective contribution of each. The C-LOGP and ACD/LogP software packages, which are commercially available, employ this fragment approach.

An extension of the fragment contribution method is the atom contribution approach, which assumes that, instead of a contribution by fragments, the value of *P*_W_^O^ can be determined by the contribution of each individual atom in the molecule. The reduced dependence on corrections is likely a major advantage of these methodologies implemented in computer software, including the well-known Ghose-Crippen, XLOGP, and VEGA-MlogP methods.

Quantum mechanical calculations have been increasingly employed to estimate the interactions between solvent and solute molecules. Predictive methods that employ charge densities and molecular electrostatic potentials with an H-bonding capability have been employed for estimating molecular hydrophobicity. Neural networks have also been employed to predict *P*_W_^O^ values from a training set of electrotopological descriptors of a large data base of drugs.

## 5. Partition Constants of Homologous Series of Antioxidants in Different Oils: Can the *P*_W_^O^ Values Determined in Octanol–Water Systems Be Employed to Predict Those in Edible Oil–Water Systems?

Frequently, researchers need to predict partition coefficients for series of homologous antioxidants where the parent compound is grafted with inert residues to modify their solubility or hydrophobicity properties. This is the case, for instance, for ascorbic acid and ascorbyl palmitate (E-300 and E-304, respectively), and for food-approved propyl, octyl, and lauryl gallates (E310, E-311 and E312, respectively). In these cases, the focus is placed on the contribution that the methylene groups makes to *P*_W_^O^ values, under the assumption that, for a given binary oil–water mixture, is constant and independent of the nature of the parent chemical, which makes a constant contribution to *P*_W_^O^.

Freiría-Gándara et al. [67] and Costa et al. [68] determined the values of the partition coefficients for the distributions of several series of homologous in oil–water binary mixtures and, for the sake of comparisons, in octanol-water. The chemical structures of the AOs employed and the determined partition constant values are displayed in Table 4.

According to the fragment approach, the contributions to *P*_W_^O^ come from three fragments. One is that of the polyphenolic moiety, the second one is that of terminal methyl group, and the third one is that from the methylene groups. Both the aromatic moiety and the terminal methyl group are the same for all individuals of each series, and thus make a constant contribution to *P*_W_^O^. However, the contribution coming from methylene groups is not constant because the different species contain a variable number of carbon atoms. Thus, the log(*P*_W_^O^) values of each series of homologous antioxidants (Table 4) can be computed as the sum of two main contributions, in Equation (5), where parameter a_O_ stands for the (constant) contribution of the non-alkyl part of the molecule to *P*_W_^O^, b_O_ stands for the contribution of each methylene group, and n_CH_2__ is the number of methylene groups in the alkyl chain of the antioxidant. This linear relationship between log(*P*_W_^O^) and the contribution of methylene groups in the series of homologous compounds is usually known as the Colander relationship [73], predicting that an increase in the number of C atoms in the alkyl chain will increase the hydrophobicity of the AOs and thus its solubility in oil [67].

(5) $$\log(P_{W}^{O}{)=a}_{O}{+b}_{O}n_{{\mathrm{CH}}_{2}}$$

**Figure 3:**
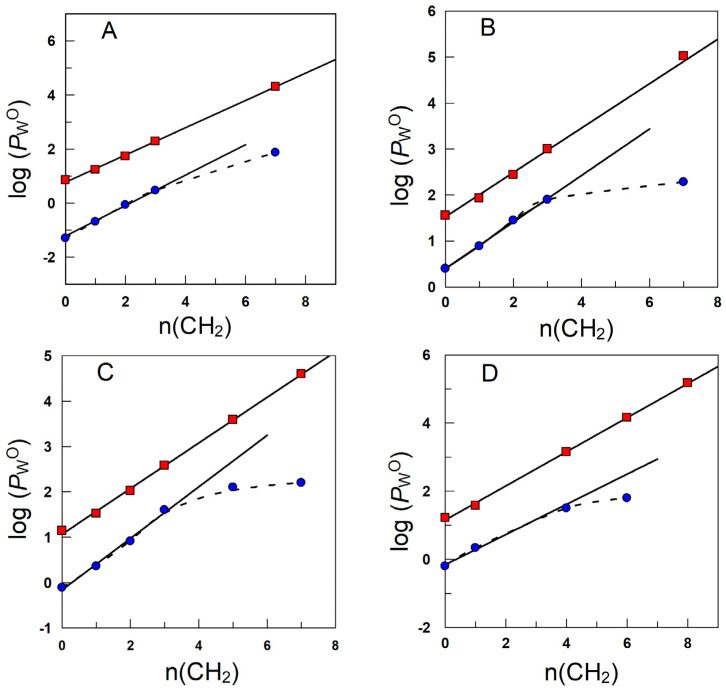
Changes in log(*P*_W_^O^) with the number of methylene groups in the alkyl chain (●) of ester derivatives of gallic (**A**), caffeic (**B**), and protocatechuic (**C**) acids and hydroxytyrosol (**D**) in olive oil–water binary systems. *P*_W_^O^ values are determined experimentally by employing the shake–flask method. For the sake of comparison, the partition coefficients of the same antioxidants in octanol–water systems (*P*_W_^OCT^, ■) are also displayed. *P*_W_^OCT^ values are estimated by employing the Molinspiration (fragment-based) computer software [74] (see Table 2 for details). Figure adapted from Freiría et al. [67] with permission, copyright American Chemical Society.

Figure 3A–D shows plots of the variations of log(*P*_W_^O^) (olive and octanol) vs. the number of -CH_2_ groups for the esters derived from gallic (GA; Figure 3A), caffeic (CA; Figure 3B), and protochateuic (PT; Figure 3C) acids and hydroxytyrosol (HT, Figure 3D), which are linear except for the most hydrophobic AOs (*n* > 7), where deviations from the linearity are evident. These curved or even two-phasic relationships are obtained when including most hydrophobic or hydrophilic compounds, because their solubility in one of the phases is rather low, yielding *P*_W_^O^ values with large errors. Similar deviations are commonly observed in the studies of homologous series of compounds and their values are not usually considered when determining the slopes and intercepts (Table 5). The goodness of fit for any particular homologous series can, however, be quantified in terms of the standard deviation of the least squares fit of these data if necessary.

The contribution of the methylene group was, as expected, very similar, no matter the oil or the AO series (differences less than 10%) considered, with an average value of b_O_ = 0.53 ± 0.02. This result should not be surprising, because the composition of the oils was quite similar (though the percentage and nature of the fatty acids may be different). Remarkably, this average value for the slope was quite similar to the average value obtained in octanol, b_oct_ = 0.50 ± 0.01, suggesting that the contribution of the methylene group to the hydrophobicity was very similar in relative apolar solvents.

The different structures for the aromatic moieties of the AOs employed made the intercepts different for each homologous series, following the order a_GA_ < a_PT_ ≈ a_HT_ < a_CA_. The non-alkyl moieties of the antioxidants contain different groups and the a_O_ values refer to different oils with different compositions, hence they cannot be compared directly to each other. However, for a given oil (e.g., olive), the comparison of the a_O_ values for GA and PT highlights the big effect of additional -OH groups on the overall hydrophobicity of the molecules. The addition of a single (HT) or double bond (CA) to the alkyl chain also has a big effect on the lipophilicity of the molecules. Further details and discussion can be found elsewhere [67,68].

Extrathermodynamic relationships between *P*_W_^O^ values in different solvents can also be established for predictive purposes [56,75] because the partition constants are, at the end, equilibrium constants, and the linear relationships between *P*_W_^O^ and *P*_W_^OCT^ with the length of the alkyl chain allows for deriving a relationship for the type ln *P*_W_^O^ = A + Bln*P*_W_^OCT^. The high relevance of *P*_W_^OCT^ for drug discovery, design, and development, means that a large number of databases containing values for this key physicochemical parameter are available in the literature. These databases include several thousands of compounds and, if for whatever reason *P*_W_^OCT^ values are not available for a particular species, it can be easily calculated with the aid of various computer programs that employ different descriptors (see Section 4.2 and Table 2 and Table 3). Thus, for predictive and practical purposes, it is was deemed interesting to investigate whether there was some kind of relationship between *P*_W_^OCT^ and *P*_W_^O^ that allowed for the prediction of *P*_W_^O^ values in edible oils from the *P*_W_^OCT^ ones.

**Figure 4:**
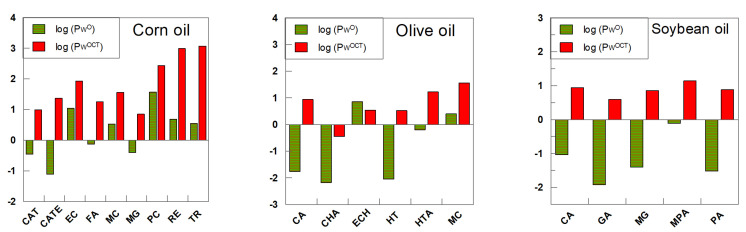
Comparison between the experimentally determined *P*_W_^O^ values in oil–water systems [76] and the theoretical *P*_W_^OCT^ values determined by employing the Molinspiration software (accessed on 23 February 2023) [74] for series of antioxidants with different molecular structures. CA: caffeic acid; CAT: catechol; CATE: catechin; CHA: chlorogenic acid; EC: ethyl caffeate; ECH: ethyl chlorogenate; FA: ferulic acid; GA: gallic acid; HT: hydroxytyrosol; HTA hydroxol acetate; MC: methyl caffeate; MG: methyl gallate; PA: protocatechuic acid; MPA: methyl protocatechuate; PC: propyl caffeate; RE: resveratrol; TR: trolox.

Figure 4 shows the experimental *P*_W_^O^ values determined for a variety of antioxidants in different oils and, for comparisons the computer estimated *P*_W_^OCT^ values for the same antioxidants. As can be observed, there were significant differences between the *P*_W_^OCT^ and *P*_W_^O^ values for the different antioxidants and oils. Such a difference can be interpreted in terms of the expected higher hydrogen bond donating capacity of octanol compared with that of common edible oils, because the main component of octanol has one hydroxyl group, meanwhile edible oils are composed of glycerol esters present in different percentages. Thus, the intermolecular forces present in, for example, octanol, should be very different in size from those in edible oils. Hence, the results in Figure 4 clearly show that *P*_W_^OCT^ values cannot be used to predict the hydrophobicity of antioxidants in edible oils and that *P*_W_^O^ values should be determined for each antioxidant and edible oil.

**Figure 5:**
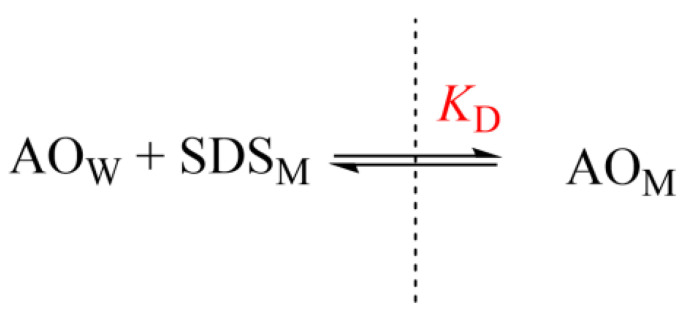
Representation of the association process of antioxidants to micellar aggregates. AO_W_ stands for the antioxidant present in the water phase, AO_M_ stands for that in the micellar phase, and SDS_M_ stands for the micellized surfactant.

Similar results can be found when considering the partitioning of antioxidants in binary systems other that oil–water. For instance, the association constant *K*_D_ of antioxidants with, for example, sodium dodecyl sulfate micelles, SDS micellar aggregates, Figure 5, can be, formally, considered similar to the oil–water partition constant *P*_w_^O^, Figure 1, because both are two-state systems where the antioxidant is located in the aqueous or in the micellar phases.

**Figure 6:**
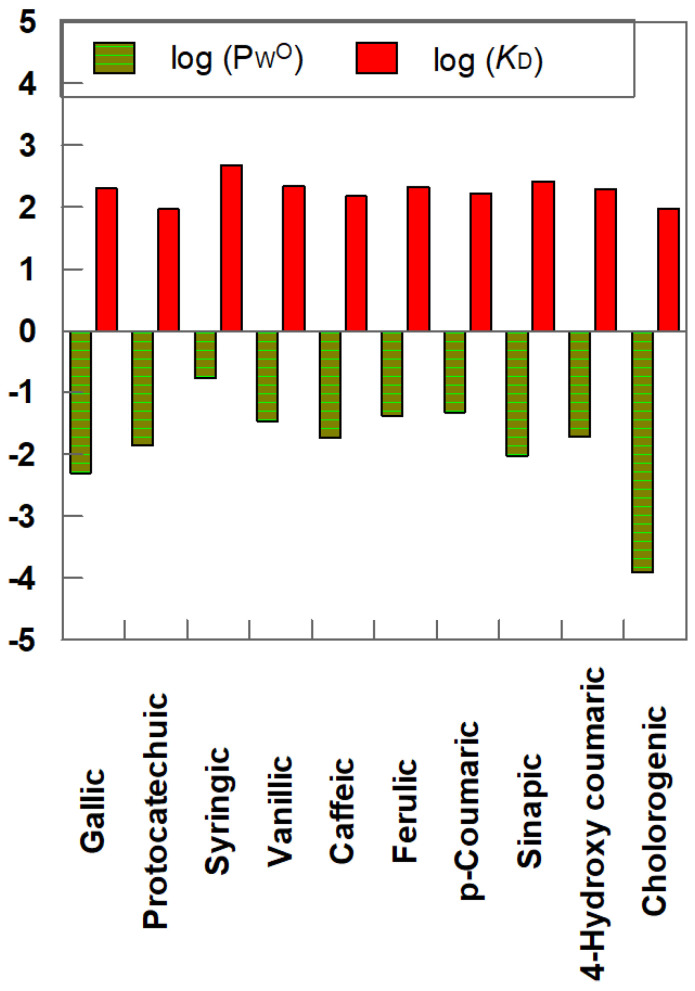
Values of experimentally determined association constants *K*_D_ between antioxidants and SDS micelles [77] (pH = 7.4, buffered solution) and the theoretically determined partition constants *P*_W_^OCT^ at the same pH for a series of phenolic acids.

Vañova et al. [77] determined, by employing micellar electrokinetic chromatography, the association constants of a series of phenolic acids between water and SDS micelles (*K*_D_), and, when comparing them with the theoretically determined octanol–water (*P*_W_^OCT^) partition constants (Molinspiration, accessed on 23 February 2023; Figure 6,) no relationship could be established between them, confirming that the values of the association constant *K*_D_ could not be predicted from the *P*_W_^OCT^ values.

In summary, the large differences between the experimentally determined *P*_W_^O^ values in oil–water systems and the theoretical *P*_W_^OCT^ values proves that, in general, *P*_W_^O^ values cannot be predicted from *P*_W_^OCT^ values and thus should be determined for each antioxidant and oil.

## 6. Effects of Acidity: Apparent Partition Coefficients of Ionizable Antioxidants

**Figure 7:**
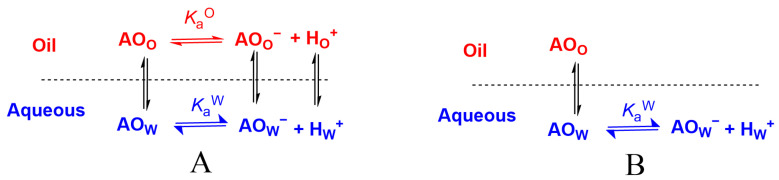
Distribution of an ionizable antioxidant AO in a biphasic oil–water system. (**A**) Assuming that ionization can take place in both phases. (**B**) Assuming that ionization only takes place in the aqueous phase. *K*_a_^W^ and *K*_a_^O^ are the ionization constant of the antioxidant in the aqueous and oil phases, respectively.

When considering phenolic acids (or any other ionizable antioxidant), one must bear in mind that ionic and neutral species exhibit different polarities and thus their intermolecular interactions with the solvent should be different [50,59,78]. The partition constant *P*_W_^O^, defined by Equation (4), only considers the concentration of neutral molecules, but if the antioxidant is partially or fully ionized under some experimental conditions (as it may occur to most phenolic antioxidants in the acidity range of most foods, pH 4–7), then *P*_W_^O^ values become pH-dependent and need to be considered as “apparent” values. These partition constants are commonly designed by *P*_W_^O^ (app), which is defined in terms of all ionized and neutral forms present at a particular pH. In principle, the antioxidants (and any other ionizable compound) can be ionized in both the aqueous and oil phases, as shown in Figure 7.

However, in general, ionization of the antioxidants in the oil phase can be neglected because the ionization constants of weak acids in oils (*K*_a_^O^) are much smaller, by 5–6 orders of magnitude, than those in the aqueous phase *K*_a_^W^ [79], so that the ionization of the solute in the oil can be neglected (p*K*_a_(O) >> p*K*_a_(W)). Thus, the distribution of the ionizable antioxidants can be simplified as indicated in Figure 7B, and the apparent partition coefficient is given by Equation (6) [67,68]. Some literature values for the ionization constants of phenolic acids are displayed in Table 6.

(6) $$P_{W}^{O}(\mathrm{app})=\frac{{(\mathrm{AO}}_{O})\;}{{(\mathrm{AO}}_{W}{)+(\mathrm{AO}}_{W}^{-})}=\frac{P_{W}^{O}}{1+\frac{K_{a}^{W}}{\left[H^{+}\right]}}$$

**Figure 8:**
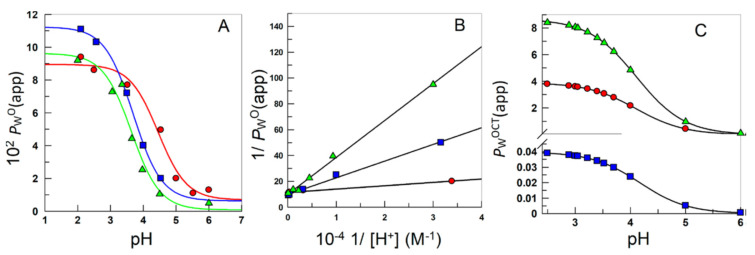
Experimental and theoretical variations of the apparent partition constants of ionizable antioxidants (gallic acid (●), ascorbic acid (■), and caffeic acid (▲)) with the acidity (pH) of the aqueous phase in olive oil–water (**A**) and linear plots of 1/*P*_W_^O^ (app) with pH according to Equation (6) (**B**). For the sake of comparisons, in octanol-water (**C**) binary systems. The solid line is the theoretical curve obtained by fitting the experimental data to a sigmoidal Henderson–Hasselbach type (Equation (6)). Simulations are done assuming that the ionization of the antioxidant in the oil phase is negligible. Figure adapted from Freiría et al. [67], with permission, copyright the American Chemical Society.

Equation (6) predicts that the variation of *P*_W_^O^ (app) with the acidity is sigmoidal, as shown in Figure 8A,C. Note that with the limit of low pH (pH at least two units below the p*K*_a_ of the ionizable antioxidant), the apparent partition coefficient approaches that of the neutral form, *P*_W_^O^, because [H^+^] >>> *K*_a_. Thus, the upper limit value of *P*_W_^O^ (app) is that corresponding to the neutral molecule (*P*_W_^O^).

In the case that, for any reason, the value of *P*_W_^O^ is unknown and/or cannot be determined experimentally, Equation (6) can be linearized so that the variation of 1/*P*_W_^O^ (app) with 1/[H^+^] should be linear from where the value of *P*_W_^O^ can be determined, as illustrated in Figure 8B.

When necessary, one can easily determine the effective local concentrations of the antioxidant in the oil and aqueous phases by bearing in mind the concentration of the antioxidant, in terms of the total volume V*_T_* of the system, using Equation (7). Note that brackets mean concentrations in moles per liter of the total volume, meanwhile parentheses indicate the effective concentrations in moles per liter of the corresponding phase.

(7) $${[\mathrm{AO}}_{T}{]V}_{T}={(\mathrm{AO}}_{oil}{)V}_{oil}+{(\mathrm{AO}}_{W}{)V}_{W}+{(\mathrm{AO}}_{W}^{-}{)V}_{W}$$

The concentration of the neutral weak antioxidant in the aqueous phase of the binary oil–water system (AO_W_) relative to the total (stoichiometric) concentration is given by the Henderson–Hasselbach type Equation (8), where Φ_O_ = V*_oil_*/(V*_oil_* + V*_water_*) and Φ_W_ = V*_water_*/(V*_oil_* + V*_water_*).

(8) $$\frac{{(\mathrm{AO}}_{W})}{{[\mathrm{AO}}_{T}]}=\frac{1}{\Phi_{W}+P_{W}^{O}\Phi_{O}+\frac{K_{a}}{{[H}^{+}]}}$$

**Figure 9:**
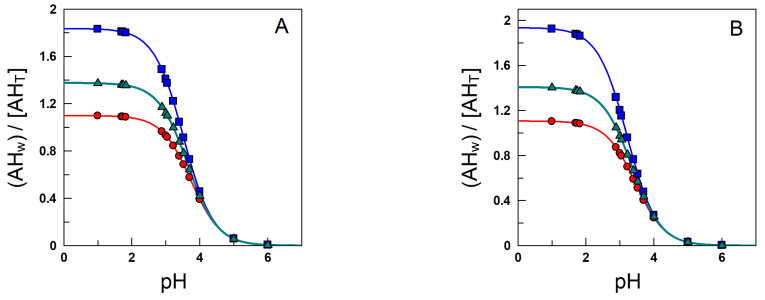
Changes in the effective concentration of the neutral forms of the antioxidants caffeic acid (**A**) and gallic acid (**B**) in the aqueous region relative to the stoichiometric one as a function of pH in 1:1 (blue), 3:7 (teal), and 1:9 (red) v:v oil–water binary mixtures. Values calculated by employing Equation (8).

Equation (8) simulates the ratio of the effective concentration of the neutral antioxidant relative to the total (stoichiometric) concentration in terms of the pH of the aqueous phase, the volume fractions of each phase (Φ_O_ and Φ_W_), the partition constant of the neutral molecule, and the ionization constant (*K*_a_) of the antioxidant, as illustrated in Figure 9. In 1:1 oil–water mixtures, the concentration in the water phase is almost twice that of the stoichiometric concentration in the high acidity limit (e.g., at low pH), meanwhile in 1:9 oil–water mixtures, the concentration of antioxidant in the water phase is very similar to the stoichiometric concentration.

In cases in which the monoprotic phenol undergoes partial ionization in the oil and aqueous phases (Figure 7A), the corresponding ionization constants are defined by Equations (9) and (10), respectively, and thus the apparent partition constant is given by Equation (11).

(9) $$K_{a}^{W}=\frac{{(\mathrm{AO}}_{W}^{-}{)(H}_{W}^{+})}{{(\mathrm{AO}}_{W})}$$

(10) $$K_{a}^{O}=\frac{{(\mathrm{AO}}_{O}^{-}{)(H}_{O}^{+})}{{(\mathrm{AO}}_{O})}$$

(11) $$P_{W}^{O}(\mathrm{app})=\frac{{(\mathrm{AO}}_{O}{)+(\mathrm{AO}}_{O}^{-})}{{(\mathrm{AO}}_{W}{)+(\mathrm{AO}}_{W}^{-})}$$

## 7. Effects of Temperature

So far, we have considered the distribution of an antioxidant between two phases at a given temperature and pressure (Figure 1). In most cases of liquid–liquid partitioning, we can neglect the effects of pressure on equilibrium partitioning, and thus here we will limit our discussion to analyze the temperature effects on the partition constant. Predicting how partition constants change with temperature is useful because the temperatures of extraction procedures and/or storage conditions may change and so we may need to be able to extrapolate values at a given temperature to other conditions. Considering relatively small temperature changes, we can safely assume, to a first approximation, that both the volumes and densities of the oil and water will remain constant, or their variations, if any, will be so small that could be considered negligible.

**Figure 10:**
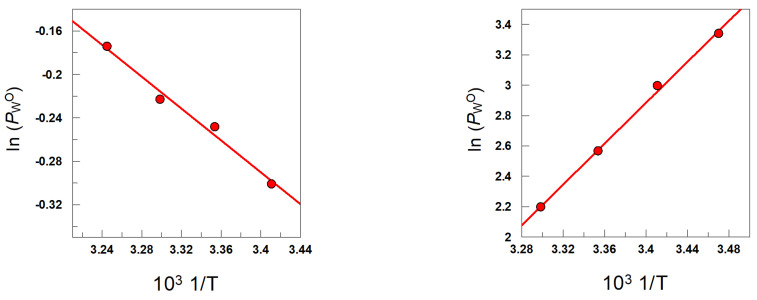
Variation of the partition constant *P*_W_^O^ of propyl gallate (PG, **left**) and α-tocopherol (TOC, **right**) with 1/*T* (K^−1^) in binary corn oil–water mixtures according to the van’t Hoff equation. Data from [83] (PG) and [84] (TOC).

From an energetic point of view, the variation in the partition constant *P*_W_^O^ of a given species between water and oil with temperature is related to thermodynamic changes, specifically the Gibbs energy, molar enthalpy, and molar entropy of the transfer of molecules from one liquid to the other. The variation in the partition constant with the temperature is given by the van’t Hoff Equation (12), where *R* stands for the universal gas constant, which predictsΔ that plots of ln *P*_W_^O^ vs. (1/*T*) should be linear, with a slope equal to Δ*H*^0,W→O^/*R*. Figure 10 shows typical van’t Hoff plots obtained for propyl gallate and α-tocopherol.

At a given temperature, the variation in the Gibbs free energy of transfer from an aqueous phase to an organic phase is given by Equation (13). According to the Gibbs equation, the Gibbs free energy Δ*G*^0,W→O^ is given by the difference between the enthalpy changes (Δ*H*^0,W→O^) and the entropy changes (Δ*S*^0,W→O^) in the partitioning process (Equation (14)), from where the entropy contribution can be determined.

(12) $$\Delta H_{{}^{T}}^{0,W\to O}=R{\left[\frac{\partial \left(\ln P_{W}^{O}\right)}{\partial \left(\frac{1}{T}\right)}\right]}_{P}$$

(13) $$\Delta G^{0,W\to O}=-RT\ln P_{W}^{O}$$

(14) $$\Delta G^{0,W\to O}=\Delta H^{0,W\to O}-T\Delta S^{0,W\to O}$$

## 8. Partitioning in Emulsions

When binary oil–water mixtures are shaken in the presence of surfactants or proteins, spherical emulsion droplets of 0.1–100 μm are formed. The surfactant is adsorbed in the oil–water interface, generating a three-dimensional, loose, boundary region of 2–20 nm thick, separating the water and oil regions that kinetically stabilize the emulsion [85,86]. The volume of this interfacial region is usually much smaller (3–5%) than the total emulsion volume, but plays a crucial role in many biological, health, and food systems. For example, oil–water emulsions are widely employed in many industries, among others, in the food, pharmaceutical, agrochemical, cosmetic, painting, printing, as well as petroleum industries. Their use as vehicles for drug delivery is an interesting approach that is currently being explored [4,30,68].

**Figure 11:**
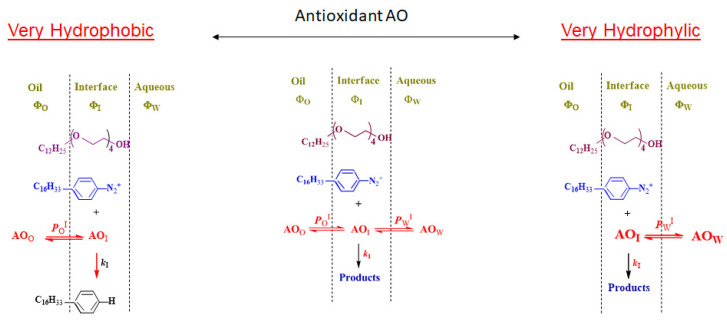
Partitioning of antioxidants in emulsions according to their polarity. In general, two partition constants are needed to define their distributions, but two limiting situations where only one partition constant is needed can be found. The first limiting situation when considering very hydrophilic antioxidants (e.g., phenolic acids) is that they are essentially oil-insoluble and only *P*_W_^I^ is needed to define their distribution. The second limiting situation is found when considering water-insoluble antioxidants (i.e., very hydrophobic antioxidants such as tocopherol). In these cases, the antioxidants only partition between the interfacial (I) and oil (O) regions and their distributions are described by the partition constant *P*_O_^I^ [88,89,90,91,92]. Figure reproduced from S. Losada-Barreiro, PhD Thesis, Universidad de Vigo, 2014, with permission.

Emulsions containing edible oils are prone to oxidation, and to minimize this undesirable reaction, antioxidants are usually added to the system. As in the binary systems, antioxidants partition thermodynamically between the aqueous, interfacial, and oil regions according to their solubility (Figure 11), and their distribution is defined by the partition constants between the water-interfacial, *P*_W_^I^, and oil-interfacial, *P*_O_^I^, regions, as shown in Equations (15) and (16), respectively [42,45,68,87].

(15) $$P_{W}^{I}=\frac{{(\mathrm{AO}}_{I})}{{(\mathrm{AO}}_{W})}$$

(16) $$P_{O}^{I}=\frac{{(\mathrm{AO}}_{I})}{{(\mathrm{AO}}_{O})}$$

Note that the ratio *P*_W_^I^/*P*_O_^I^ = (AO_O_)/(AO_W_) = P_W_^O^. Thus, *P*_W_^I^/*P*_O_^I^ is numerically equal to the partition constant in a binary oil–water system because it can be considered as a limiting case of oil-in-water emulsions where no surfactant is added. This means that the partition constants in multiphasic systems cannot be extrapolated from that in the simpler binary system, and need to be determined in the intact emulsions to avoid disruptions of the existing equilibria. We also want to note that the partition constants, defined by Equations (15) and (16), do not consider the size of the droplets in the emulsion, i.e., it is assumed that they are independent of the size of the droplets. The point has been experimentally proven by determining the distributions of various antioxidants in emulsions containing droplets of different size (macro and nano-emulsions): no changes in the partition constants were detected [41,42,44,45,93,94,95]. Table 7 lists some partition constants for common antioxidants determined in intact emulsions prepared with edible oils under different experimental conditions. Details on the methodology employed and experimental conditions can be found elsewhere and in the references therein [41,42,44,45].

**Figure 12:**
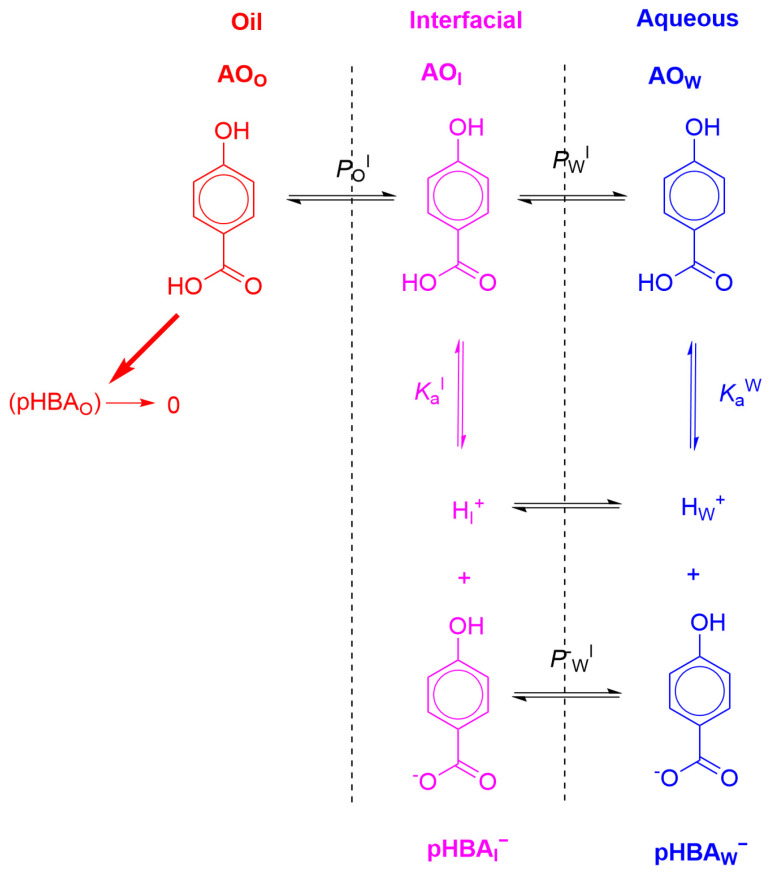
Equilibria for the distribution of a phenolic acid, AO, in emulsions. Phenolic acids only ionizes significantly at the typical acidities (pH = 2–6) of the emulsified foods. Adapted from Losada-Barreiro et al. [78] with permission, copyright J. Wiley and Sons.

As in the case of binary systems, the distribution of ionizable antioxidants (e.g., phenolic acids) also depends on the acidity of the aqueous phase, and their ionization equilibrium needs to be taken into consideration (Figure 12). Both neutral and ionized species may be distributed between the interfacial and aqueous regions, but, as in binary systems, the solubility of ionized species in the oil region is negligible. In these cases, two apparent partition constants need to be considered, *P*_O_^I^ (app) and *P*_W_^I^ (app), defined by Equations (17) and (18), respectively [41,42,68].

(17) $$P_{O}^{I}(\mathrm{app})=\frac{{(\mathrm{AO}}_{I}{)+(\mathrm{AO}}_{I}^{-})}{{(\mathrm{AO}}_{O})}$$

(18) $$P_{W}^{I}(\mathrm{app})=\frac{{(\mathrm{AO}}_{I}{)+(\mathrm{AO}}_{I}^{-})}{{(\mathrm{AO}}_{W}{)+(\mathrm{AO}}_{W}^{-})\;}$$

Losada-Barreiro et al. [90] investigated the driving force for the hydrophobic effect that makes antioxidants partition between the oil, aqueous, and interfacial regions of oil-in-water emulsions. They determined the Gibbs free energy, enthalpy, and entropy values for the transfer of hydrophilic and hydrophobic antioxidants from water to the interfacial and from oil to the interfacial regions of emulsions by employing Equations (19)–(22).

(19) $$\Delta G_{T}^{0,\;W\to I}=RT\ln\frac{V_{m}^{W}}{P_{W}^{I}V_{m}^{I}}$$

(20) $$\Delta G_{T}^{0,\;O\to I}=RT\ln\frac{V_{m}^{O}}{P_{O}^{I}V_{m}^{I}}$$

(21) $$\Delta H_{T}^{0,W\to I}=R{\left[\frac{\partial \left(\ln P_{W}^{I}\right)}{\partial \left(\frac{1}{T}\right)}\right]}_{P}$$

(22) $$\Delta H_{T}^{0,O\to I}=R{\left[\frac{\partial \left(\ln P_{O}^{I}\right)}{\partial \left(\frac{1}{T}\right)}\right]}_{P}$$

**Figure 13:**
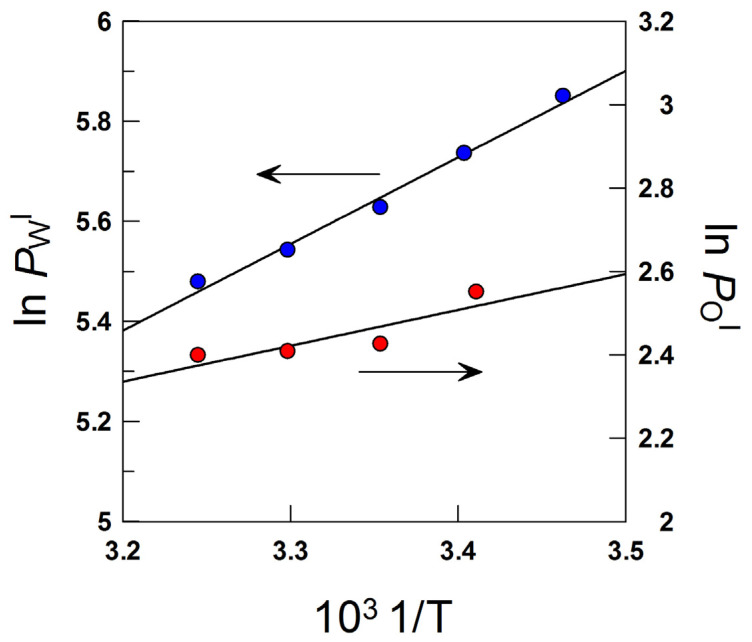
Variations of ln*P*_O_^I^ and ln*P*_W_^I^ with 1/*T* (Kelvin) according to the van’t Hoff equation for caffeic acid (blue) and α-tocopherol (red) in corn oil emulsions. Figure reproduced from Losada-Barreiro et al. [90] with permission, copyright Royal Society of Chemistry.

The enthalpy and entropy changes for the transfer of CA from the aqueous to the interfacial region, $\Delta H_{T}^{0,W\to I}$ and $\Delta S_{T}^{0,W\to I}$, can be obtained from the variations of *P*_W_^I^ at a series of temperatures by using the van´t Hoff Equation (12) and the Gibbs Equation (14), respectively. Similar equations are employed to determine $\Delta H_{T}^{0,O\to I}$ and $\Delta S_{T}^{0,O\to I}$ for the transfer of TOC. Figure 4 illustrates the variation of ln(*P*_W_^I^) and ln(*P*_O_^I^) with 1/*T* according to the van´t Hoff equation, from where the enthalpy values of the transfer are obtained. An illustrative example of the determination of the thermodynamic parameters in emulsions is given in Figure 13 for caffeic acid and tocopherol.

## 9. Final Remarks and Conclusions

The goal of this manuscript was to have a first glimpse at the partitioning behavior of antioxidants between two immiscible liquids and of the molecular interactions that govern their partitioning, showing that simple thermodynamic concepts, in particular, chemical potential, can be used to quantify equilibrium partitioning. The interest in partitioning comes, in part, because the massive production of agri-food wastes and by-products generated in the agri-food industrial sector not only present safe disposal issues, but also contribute negative environmental impacts. This environmental stress is enormous in our globalized world, and a sustainable utilization of agri-food waste and/or by-products to produce value-added products for potential applications in cosmetic, pharmaceutical, or food industrial uses can provide considerable opportunities for earning an additional income for the dependent industry. In addition, adding economic value to environmentally undesirable agriculture and/or food wastes contributes to ensuring sustainable food production and security. To date, most of these wastes have been employed as organic fertilizers or as source of fuel, but modern technologies in combination with “green chemistry” principles lead to the effective and rational use of wastes and by-products producing value-added materials.

Bioactive components such as phenolic antioxidants, isolated from fruits, vegetables, and their wastes include polyphenols, tannins, flavonoids, and a variety of vitamins. In the marine sector, polyphenols with an antioxidant activity can be obtained from the fish and shellfish industries and from natural or harvested seaweeds. Interest in polyphenolic antioxidants has also increased notably because of their protective roles in food and pharmaceutical products against oxidative deterioration and in the body and against oxidative stress-mediated pathological processes. Indeed, the search for natural antioxidants in plants and plant-derived compounds, as well as their use in foods, cosmetics, pharmacological formulations, parenteral emulsions, etc., requires appropriate methods to determine their distributions in order to reveal their mechanisms of activity and to understand the kinetics of their reactions with free radicals.

Nevertheless, the valorization of wastes and by-products requires extraction procedures where the partition constants between two solvents play a major role. Determining partition constants has received much attention in the assessment of relative lipophilicity and hydrophilicity of a compound because it dictates formulation strategies for various biological, medical, and pharmaceutical processes. It is also important for synthesis and purification, and for understanding the release and distribution of drugs in human body. *P*_W_^O^ values are commonly used for the description of the hydrophobicity or lipophilicity of compounds and some of them, for example, the *P*_W_^OCT^ values are, in general, good descriptors for the characterization of the relationship between the structure and biological, pharmacological, and ecological effects of various drugs. It is not surprising, therefore, the enormous interest in determining the distributions of antioxidants in binary oil–water systems as they are one of the secondary metabolites present in nature. Their distribution (and in general, that of any other compound) depend on the balance of all intermolecular forces, mainly hydrogen bonding and van der Waals interactions between the solute and oil and water phases. Because the composition of the various alcohols commonly employed to mimic the partitioning of drugs in the human body (octanol, hexanol, etc.) is quite different from that of edible oils, the partition constant values determined in oil cannot be extrapolated to others, and hence need to be determined individually.

## Figures and Tables

**Table 1:** Summary of the major intermolecular forces. For the sake of energetic comparisons, the interionic ion–ion force is also included.

Type of Force	Energy (kJ/mol)	Interacting Species
Ionic bond	300–600	Ions/Ions
Hydrogen bonding	20–40	Polar molecules containing N-H, O-H, or F-H, the link is a shared H atom
Ion–Dipole	10–20	Ion/polar molecule
Dipole–Dipole (Keeson)	1–5	polar molecule/polar molecule
Dipole–Induced dipole (Debye)	2–10	Stationary polar molecules/all types of molecules
Induced dipole–Induced dipole (London dispersion forces)	<2	All types of molecules/all types of molecules

**Table 2:** Some current experimental and computational methods employed to estimate partition constant values in oil–water and in octanol–water binary systems.

Experimental			
Method	Advantage	Weakness	Applicability
Shake-flask	Most realistic, reliable, low experimental demand	Time consuming, large amounts of mutually saturated solvents required, emulsification may be a problem	Molecules of moderate hydrophobicity. Usually not recommended for very hydrophobic or hydrophilic compounds and when the tested substance dissociates
Slow-stirring methods	Avoid formation of emulsions	Time consuming, requires large amounts of solvent and product	Similar to the shake-flask method
Reverse-phase chromatography	Rapid, does not require large amounts of product nor solvent	Poor reproducibility because of different retention mechanisms, requires HPLC instrumentation	
Micellar electrokinetic chromatography	Good agreement with shake-flask method		Applicable to ionic substances
Filter probe methods	Rapid	Expensive lab set-up, time consuming	
Software packages			
Name	Company	Freeware	Comments
ACD/logD	Advanced Chemistry Development (www.acdlabs.com)	No	Fragment-based
ADMET predictor	Simulation Plis Inc. (www.simulationsplus.com)	No	Neural network
AlogP	Virtual Computation Chemistry Laboratory (www.vcclab.org)	Yes	Neural network
Hyperchem	Hypercube Inc. (www.hypercube.com)	No	Atom-additive method
MolInspiration	Molinspiration Cheminformatics, (https://www.mlinspiration.com)	Yes	Fragment based
SPARC	Univeristy of Georgia (http://www.ibmlc2.chem.uga.edu/sparc/)	Yes	Allows calculations under different ionic strength conditions

**Table 3:** Examples of simple one-parameter linear free energy relationships (LFERs) proposed for connecting partition constants in various two-phase systems.

Partition Constant/Coefficients Correlated	LFER
Octanol–water (oct–w)/aqueous solubility (sat)	$\log K_{i,w}^{\mathrm{oct}}=-a\log K_{i,w}^{\mathrm{sat}}+b$
Organic carbon–water (oc–w)/octanol–water (oct–w)	$\log K_{i,w}^{\mathrm{oct}}=-a\log K_{i,w}^{\mathrm{oct}}+b$
Lipid–water (lip–w)/octanol–water (oct–w)	$\log K_{i,w}^{\mathrm{lip}}=-a\log K_{i,w}^{\mathrm{oct}}+b$

**Table 4:** Experimental partition constants for the distributions of AOs of different hydrophobicities in oil–water systems and theoretical octanol–water values, *T* = 25 °C. Data extracted from Freiría-Gándara et al. [67].

ANTIOXIDANT			Log *P*_W_^O^			
Structure	-R	*n*(CH_2_)	Olive	Soybean	Corn	Octanol
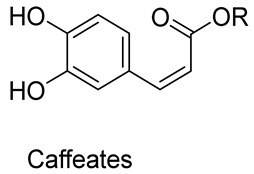	-CH_3_ (C1)	0	0.40	0.45	0.52	1.56
	-CH_2_ CH_3_ (C2)	1	0.89	0.92	1.04	1.93
	-(CH_2_)_2_ CH_3_ (C3)	2	1.45	1.48	1.58	2.44
	-(CH_2_)_7_ CH_3_ (C8)	7	2.23	2.25	2.81	5.02
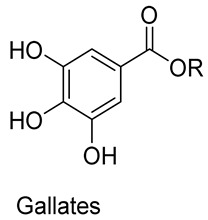	-CH_3_ (C1)	0	−1.30	−1.40	−0.94	0.85
	-CH_2_ CH_3_ (C2)	1	−0.70	---	−0.60	1.23
	-(CH_2_)_2_ CH_3_ (C3)	2	−0.07	−0.07	0.10	1.73
	-(CH_2_)_3_ CH_3_ (C4)	3	0.48	0.51	0.60	2.29
	-(CH_2_)_7_ CH_3_ (C8)	7	1.88	2.29		4.31
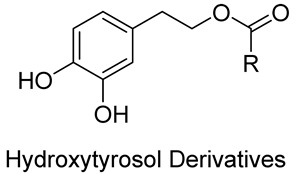	CH_3_ (C2)	0	−0.20	---		1.22
	CH_2_ CH_3_ (C3)	1	0.34	---		1.58
	(CH_2_)_4_ CH_3_ (C6)	4	1.50	---		3.15
	(CH_2_)_6_ CH_3_ (C8)	6	1.61	---		4.16
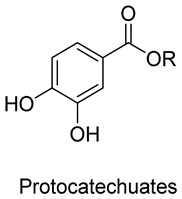	-CH_3_ (C1)	0	−0.11	−0.11		1.14
	-CH_2_ CH_3_ (C2)	1	0.27	0.36		1.52
	-(CH_2_)_2_ CH_3_ (C3)	2	0.91	0.91		2.02
	-(CH_2_)_3_ CH_3_ (C4)	3	---	1.60		2.58
	-(CH_2_)_5_ CH_3_ (C6)	5	1.72	1.71		3.59

**Table 5:** Slopes and intercepts for the variations of log(*P*_W_^O^) with the number of methylene groups according to Equation (5) (Figure 3) for ester derivatives of different antioxidants such as gallic acid (GA), caffeic acid (CA), protocatechuic acid (PT), and hydroxytyrosol (HT). Values from Freiría-Gándara et al. [67].

OIL	Antioxidants	Gallic	Caffeic	Protocatechuic	Hydroxytyrosol
Octanol (OCT)	a_OCT_	0.77 ± 0.03	1.53 ± 0.05	1.06 ± 0.03	1.15 ± 0.04
	b_OCT_	0.51 ± 0.01	0.49 ± 0.01	0.50 ± 0.01	0.50 ± 0.01
Olive (OL)	a_OL_	−1.15 ± 0.04	0.40 ± 0.03	−0.18 ± 0.07	−0.16 ± 0.02
	b_OL_	0.50 ± 0.01	0.51 ± 0.01	0.55 ± 0.04	0.44 ± 0.02
Soybean (SO)	a_SO_	−1.18 ± 0.06	0.44 ± 0.03	---	---
	b_SO_	0.58 ± 0.03	0.52 ± 0.03	---	---
Corn (CO)	a_CO_	−1.03 ± 0.10	0.52 ± 0.01	---	---
	b_CO_	0.54 ± 0.06	0.53 ± 0.01	---	---

**Table 6:** Acidity constants for some phenolic acids for the carboxylic acid [p*K*a(1)] and that for the -OH group in the *p*-position [(p*K*a(2)].

Phenolic Acid	Molecular Structure	p*K*_a_(1)	p*K*_a_(2)	Ref
p-Coumaric	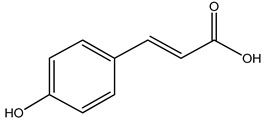	4.37	9.20	[80]
Caffeic	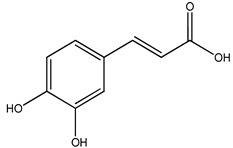	3.94 4.30	8.47 8.51	[80] [81]
Ferulic	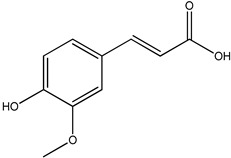	4.50 4.30	9.21 8.81	[80] [81]
3,4 Dihydroxy phenylacetic	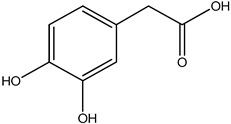	3.21	9.33	[80]
Gallic acid	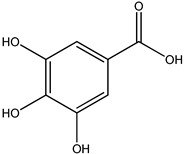	4.10 4.11	8.38 8.47	[80] [81]
Vanillic	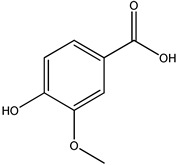	4.58 4.17	9.39 8.81	[80] [81]
p-Hydroxybenzoic	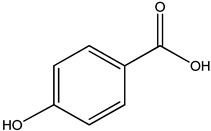	4.40 4.26	9.54 8.84	[80] [81]
Trolox	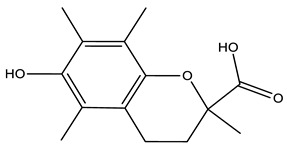	3.89	11.92	[82]

**Table 7:** Values of the partition constants of antioxidants (AO) between the aqueous-interfacial (*P*_w_^I^) and oil-interfacial (*P*_O_^I^) regions of emulsions. Partition constant values are determined in the intact emulsions by employing a kinetic methodology [42,45] to avoid disruption of the existing equilibria to avoid biasing of values. TW20 = Tween 20, TW80 = Tween 80, SP80 = Span 80. BG: butyl gallate; CA: caffeic acid; CAT: catechol; CGA: chlorogenic acid; CATE: catechin; DCG: dodecyl chlorogenate; EC: ethyl caffeate; ECG: ethyl chlorogenate; EG: ethyl gallate; EHT: hydroxytyrosol ethanoate; GA: gallic acid; HET: hydroxytyrosol hexanoate; HHT: hydroxytyrosol hexadecanoate; HT: hydroxytyrosol; HTA hydroxytyrosol acetate; LCG: lauryl chlorogenate; LG: lauryl gallate; LHT: hydroxytyrosol laurate; MC: methyl caffeate; MG: methyl gallate; OC: octyl caffeate; OCG: octyl chlorogenate; HCG: hexadecyl chlorogenate; OG: octyl gallate; OHT: hydroxytyrosol octanoate; PC: propyl caffeate; PCG: propyl chlorogenate; PG: propyl gallate; RES: resveratrol; TOC: α-tocopherol; TR: trolox.

Antioxidant	Surfactant	Oil/Water Ratio (v:v)	pH	*P*_w_^I^	*P*_O_^I^	Reference
Fish oil						
GA	TW80	4:6	3.7	118	---	[95]
GA	TW80	1:9	3.0	85	---	[96]
EG	TW80	4:6	3.7	233	706	[95]
PG	TW80	1:9	3.0	154	101	[96]
BG	TW80	4:6	3.7	559	253	[95]
OG	TW80	4:6	3.7	---	183	[95]
LG	TW80	4:6	3.7	---	142	[95]
HT	TW80	1:9	3.7	34	---	[93]
EHT	TW80	1:9	3.7	207	115	[93]
HET	TW80	1:9	3.7	---	89	[93]
OHT	TW80	1:9	3.7	---	119	[93]
LHT	TW80	1:9	3.7	---	97	[93]
HHT	TW80	1:9	3.7	---	75	[93]
Corn oil						
CATE	TW20	4:6	2.1	368	---	[97]
CAT	TW20	4:6	3.7	57	170	[98]
CA	TW20	4:6	3.7	268		[99]
PG	TW20	1:9	3.7	204	242	[100]
OG	TW20	1:9	3.7	---	29.8	[100]
LG	TW20	3:7	3.7	---	16.5	[100]
RES	TW20	4:6	2.1	4076	860	[101]
TOC	TW20	1:9	3.7	---	11.3	[90]
Olive oil						
HT	TW20/SP80	1:9	3.7	120	---	[102]
HTA	TW20/SP80	1:9	3.6	204	331	[102]
TR	TW20	4:6	2.2	5371	1773	[103]
CGA	TW20	4:6	3.7	40	---	[104]
ECG	TW20	4:6	3.7	78	---	[104]
PCG	TW20	4:6	3.7	141	---	[104]
OCG	TW20	4:6	3.7	---	111	[104]
DCG	TW20	4:6	3.7	---	124	[104]
LCG	TW20	4:6	3.7	---	159	[104]
HCG	TW20	4:6	3.7	---	89	[104]
Soybean oil						
GA	TW20	1:9	3.0	298		[105]
MG	TW20	1:9	3.0	329	---	[105]
PG	TW20	1:9	3.0	401	474	[105]
BG	TW20	1:9	3.0	789	243	[105]
OG	TW20	1:9	3.0	33	---	[105]
LG	TW20	1:9	3.0	23	---	[105]
CA	TW20	4:6	3.7	104	---	[106]
MC	TW20	4:6	3.7	445	150	[106]
EC	TW20	4:6	3.7	1355	159	[106]
PC	TW20	4:6	3.7	4727	164	[106]
OC	TW20	4:6	3.7	---	216	[106]

## Data Availability

Not applicable.
